# Graft Polymerization of Stearyl Methacrylate on PET Track-Etched Membranes for Oil–Water Separation

**DOI:** 10.3390/polym14153015

**Published:** 2022-07-26

**Authors:** Arman B. Yeszhanov, Indira B. Muslimova, G. B. Melnikova, A. S. Petrovskaya, Aibek S. Seitbayev, S. A. Chizhik, Nariman K. Zhappar, Ilya V. Korolkov, Olgun Güven, Maxim V. Zdorovets

**Affiliations:** 1 L.N. Gumilyov Eurasian National University, Satpaev Str., 5, Nur-Sultan 010008, Kazakhstan; arman_e7@mail.ru (A.B.Y.); bazarbaykyzy@list.ru (I.B.M.); galachkax@gmail.com (G.B.M.); kena3991@mail.ru (A.S.S.); 2 The Institute of Nuclear Physics, Ibragimov Str., 1, Almaty 050032, Kazakhstan; 3 A.V. Luikov Heat and Mass Transfer Institute of the National Academy of Sciences of Belarus, P. Brovki Str., 15, 220072 Minsk, Belarus; korolkovelf@gmail.com (A.S.P.); chizhik@presidium.bas-net.by (S.A.C.); 4 LLP “EcoSave”, 3 Microdistrict-9, Stepnogorsk, Akmola Region 021500, Kazakhstan; nariman_zhappar@mail.ru; 5 Department of Chemistry, Hacettepe University, Beytepe, Ankara 06800, Turkey; guven@hacettepe.edu.tr; 6 Ural Federal University, Mira Str. 19, 620002 Ekaterinburg, Russia

**Keywords:** ion-track membranes, hydrophobic modification, photo-induced graft polymerization, water–oil separation

## Abstract

In this article, results of PET track-etched membranes (PET TeMs) hydrophobized by photo-induced graft polymerization of stearyl methacrylate (SM) inside the pores were presented. The effects of monomer concentration, time of irradiation and the nature of the solvent on the degree of grafting and membrane morphology were investigated. The PET TeMs with pore diameters ranging from 350 nm (pore density of 1 × 10^8^ pore/cm^2^) to 3.05 µm (pore density of 1 × 10^6^ pore/cm^2^) were hydrophobized and tested for oil–water separation by using hexadecane–water and chloroform–water emulsions. Studies have shown high separation performance for membranes (up to 1100 mL/m^2^·s) with large pore diameters while achieving a high degree of purification.

## 1. Introduction

Water has been one of the most indispensable commodities used by all kinds of industries. During the manufacturing, separation and purification processes in petrochemical, pharmaceutical, metallurgical, oil, and gas industries large volumes of oily wastewater are being generated. Frequent oil spill accidents further make the pollution of seawater an environmental catastrophe that requires urgent remedial actions. Pollution of aquatic sources by oil has become one of the most important problems, causing not only severe environmental pollution but also threatening human health [1,2]. Separating oil and water is therefore a worldwide challenge, and extensive efforts are being made to find better solutions to this problem.

There are several physical and chemical methods for the purification of oily wastewater. A number of techniques such as solvent extraction, microwave irradiation, oxidation, landfill, electrokinetics and froth flotation have been developed to separate oil–water mixtures but with limited success either due to their low efficiency or high cost [3].

Membrane-based filtration techniques with high efficiency, low energy requirements, simplicity of operation and relatively low cost seem to be one of the most promising techniques to mitigate the oil–water separation problems [4,5,6]. Reverse osmosis [7], ultra-[8,9] and nanofiltration [10,11], membrane distillation and other types are often used to solve the problems of separation of oil–water emulsions [12,13]. Less attention has been devoted, however, to the use of track-etched membranes (TeMs) to design and develop novel membrane materials for the treatment of oily wastewater. 

TeMs are characterized by a regular pore geometry with the possibility of controlling them per unit area, as well as a narrow pore size distribution, which should have a positive effect on separation selectivity. The functionalization of pore surfaces by graft copolymerizing with polymers carrying special functions increases their use as thin film electrodes [14], as catalysts [15] and for a variety of separation needs [16,17]. The applicability of such membranes is quite wide and is used in the pharmaceutical and chemical industries; in microelectronics, as well as in the processes of ultra-, microfiltration and membrane distillation [18,19,20,21].

Graft polymerization can be implemented by using the methods of thermo-, radiation-, plasma- and photo-initiation [21,22,23]. The method of photoinitiated graft polymerization, unlike other methods of modifying the surface of a number of polymers, does not significantly affect the substrate and change its mechanical and operational properties. Since the radiation energy is low, grafting takes place under mild conditions and at low temperatures. Photoinitiated graft polymerization is widely used to modify the surface of various polymers, and it is based on the formation of radicals on the polymer surface with the help of photoinitiators that generate radicals under UV irradiation or sensitizers that are capable of abstracting a hydrogen atom from the polymer chain, and then the formed radicals initiate polymer chain growth from the surface [24,25]. 

In our recent preliminary work [26], we have shown that PET TeMs modified with hydrophobic silanes could be used for oil–water separation. However, unsatisfied results on fluxes were obtained. One of the solutions to this problem is to increase the pore diameter of membranes and develop methods for hydrophobing membranes with large pore diameters. On one hand, stearyl methacrylate (SM) is a long side chain monomer with hydrophobic properties that can be used to improve the water-repellent properties of materials [22]. On the other hand, there are currently no articles of SM being grafted to PET TeMs. At the same time, its accessibility, low price and high hydrophobic properties (up to 149° [22]) make it an excellent monomer for imparting stable hydrophobic properties to PET TeMs with large pore diameters up to 3 µm. In this article, we have applied the method of photo-induced graft polymerization of stearyl methacrylate (SM) on PET TeMs. Hydrophobized PET TeMs with different pore sizes were tested in the separation of oil-water emulsions using the model systems hexadecane–water and chloroform–water.

## 2. Materials and Methods

### 2.1. Materials

Sodium hydroxide, benzophenone, N’N-dimethylformamide, 2-propanol, stearyl methacrylate, chloroform and hexadecane were supplied by Sigma-Aldrich (Darmstadt, Germany). Stearyl methacrylate was purified from stabilizers by passing through alumina in chromatographic column. Deionized water (18.2 MΩ) was used in all experiments.

### 2.2. Preparation and Modification of Track-Etched Membranes (TeMs)

PET TeMs with pore densities of 1 × 10^8^ pores/cm^2^ (pore size is 350 nm) and 1 × 10^6^ pores/cm^2^ (pore size is around 3000 nm) were obtained by irradiation of thin PET films with Kr ions using the accelerator DC-60 (Astana branch of Institute of Nuclear Physics) with an energy of 1.8 MeV/nucleon followed by chemical treatment in 2.2 M NaOH [27]. 

**Figure 1:**
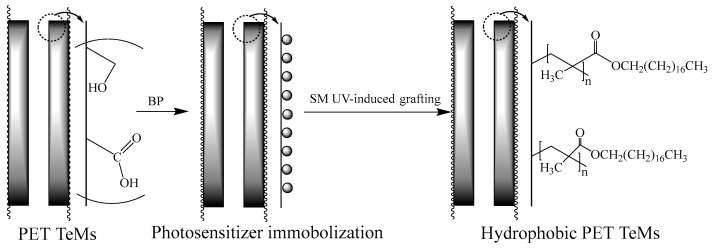
Scheme of PET TeMs hydrophobization.

Hydrophobization of PET TeMs was achieved by photo-induced graft polymerization as presented schematically in Figure 1. The method of photoinitiated graft polymerization, unlike other methods of modifying the surface of a number of polymers, does not significantly affect the substrate and change its mechanical and operational properties. Since the radiation energy is low, grafting takes place under mild conditions and at low temperatures.

The PET TeMs were first immersed in a 5% initiator solution (benzophenone) in DMF for 24 h to immobilize the photosensitizer on the membrane surface [28]. Since DMF is able to swell PET, BP could be introduced into the subsurface layer of the polymer. Then membranes were placed in a solution of stearyl methacrylate (SM) in 2-propanol with concentration range of 1–45% and irradiated under the UV-lamp OSRAM Ultra Vitalux E27 (UVA: 315–400 nm, 13.6 W; UVB: 280–315 nm, 3.0 W) for 30–120 min. Then, SM grafted PET TeMs were thoroughly washed in 2-propanol, acetonitrile, dried and weighed to determine the grafting degree by the following equation:

(1) $$\eta =\frac{\left(m_{2}-m_{1}\right)}{m_{1}}100\%$$

where *m*_1_—the weight of the membrane before grafting, *m*_2_—the weight of the membrane after grafting. To remove electrostatic charge, antistatic ionizer was used before weighing.

### 2.3. Methods of Characterization

FTIR spectra were recorded using FTIR spectrometer InfraLUM FT-08 (Lumex, Russia) with ATR accessory (PIKE, Madison, WI, USA) for determining the functional groups before and after modification. The measurements were carried out in the range from 400 to 4000 cm^−1^, 32 scans with 2 cm^−1^ resolutions.

The water and diiodomethane contact angles (CA) ±0.10 were determined on a DSA 100E (KRUSS, Hamburg, Germany) by the sessile drop method. The values of free surface energy (γ) and its specific polar component (γp) were calculated by using the respective CA values following the method described by **O**wens, **W**endt, **R**abel, and **K**aelble (OWRK) [29]. Scanning electron microscope JEOL JSM-7500F, and atomic force microscope Smart SPM-1000 AIST-NT (ALC «Microtestmachines», Gomel, Republic of Belarus_ (with tip radius not exceeding 10 nm) were used for the evaluation of pore diameters and characterization of morphology after PET TeMs modification. Gas permeability test was used to estimate effective pore sizes of the membranes at a pressure drop of 20 kPa [30].

### 2.4. Performance of Membranes in Oil–Water Separation

Separation of oil–water mixtures by hydrophobized PET TeMs was conducted by filtration and carried out according to the scheme presented in our previous work [26]. The model of oil–water emulsion was prepared using Disperser IKA T18 digital Ultra-Turrax (chloroform/water = 10:1 (vol.), hexadecane/water = 100:1 (vol.)). When the water-in-oil emulsion was filtered through the membrane, water was removed and the cleaned oil (chloroform or hexadecane) was collected in a beaker.

The flux (*F*) of the filtered oil–water mixture was calculated using Equation (2) as follows:

(2) $$F=\frac{V}{S\cdot t}$$

where *V* is the volume of the oil that permeates through the membrane, *S* is the filtration area of PET TeMs, *t* is the flow time.

The volume of water collected after separation was measured and the separation efficiency (*R*, %) was calculated using the following Equation (3):

(3) $$R=\frac{V_{2}}{V_{1}}100\%\;$$

*V*_1_ is the volume of water in water-in-oil emulsion before separation; *V*_2_ is the volume of water collected after separation.

## 3. Results

**Figure 2:**
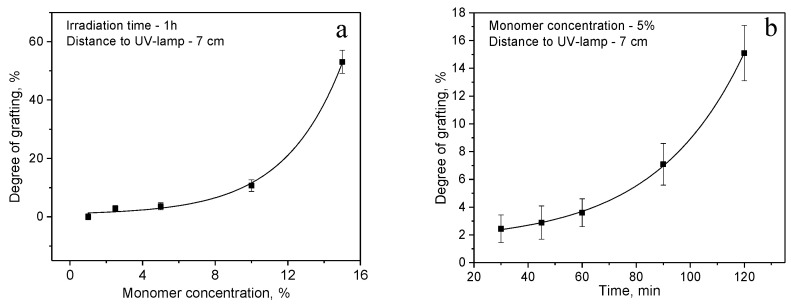
Effect of monomer concentration (**a**) and time of UV irradiation (**b**) on the degree of grafting on PET TeMs (pore diameter of initial PET TeMs is 350 nm, pore density is 1 × 10^8^ pore/cm^2^).

Figure 2a,b shows the dependence of the monomer concentration and time of UV irradiation on the degree of grafting (the distance from the UV source is constant, 7 cm). The degree of grafting increases with increasing monomer concentration, at a concentration of 15% a sharp increase in the degree of grafting was observed, however, accompanied by blockage of the channels, as can be seen from the gas permeability data presented in Table 1. Irradiation time affects the degree of grafting to a lesser extent than the concentration, so there is an increase in the degree of grafting with increasing time from 2.44% (30 min) to 15.10% (120 min) at constant monomer concentration of 5%. Under the same conditions, graft polymerization was carried out for membranes with large pore diameter of 3.05 µm and pore density of 1 × 10^6^, results are also shown in Table 1. A similar trend in grafting was observed for membranes with pore diameter of 350 nm.

Results on effective pore diameter dependence on grafting conditions are collected in Table 1, which shows that pore diameters decrease continuously with the degree of grafting. Table 2 presents the results of CA (water and diiodomethane) measurements, surface free energy (γ) and its specific polar components (γ_p_) calculated by using the work method. It is clearly seen that with increasing grafting time up to 60 min, hydrophobic properties of the membrane increase. A further increase in grafting time leads to a slight decrease in CA (water) accompanied by an increase in the specific polar component. This may be due to competing processes of PET membrane photodegradation. Indirectly, we observe this by changing the burst strength characteristics of membranes as follows: 278 kPa—for initial PET TeMs, 133 kPa—after 60 min grafting, 21.5 kPa—after 120 min grafting. In order to exploit this behavior, PET TeMs were also UV-irradiated in grafting solvent but in the absence of monomer. There was also a decrease in the burst strength to 105.9 kPa after 60 min of UV irradiation and to 19.9 kPa after 120 min, which shows that photodegradation is mainly responsible for the decreases in burst strength during grafting. This result also implies that in UV-induced grafting on PET, grafting should be carried out at as low a dose as possible in order to protect the substrate. Under similar conditions, PET TeMs with pore diameters of 350 nm, 2.10, 2.50, 2.8 and 3.05 µm were also modified by SM grafting.

**Figure 3:**
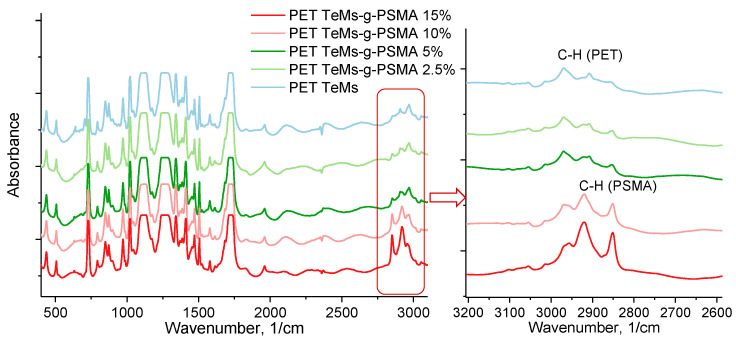
FTIR spectra of original PET TeMs in comparison with SM grafted PET TeMs at different concentrations of monomer.

The FTIR spectra of the original and modified PET TeMs are shown in Figure 3. The unmodified PET TeMs show characteristic absorption bands at 2970 cm^−1^ (benzene ring, CH), 2912 cm^−1^ (aliphatic CH), 1713 cm^−1^ (C=O group), 1615, 1470, 1430, 1409 cm^−1^ (aromatic vibrations of the carbon skeleton), 1340 cm^−1^ (O-CH), 1238 cm^−1^ (vibrations of bonds of ether groups C(O)-O), 970 cm^−1^ (O-CH_2_). SM grafting leads to the appearance of new peaks at ~2852, 2925 and 2951 cm^−1^, related to the stretching vibrations of the C-H bonds of long side chains of the grafted polymer [31,32].

**Figure 4:**
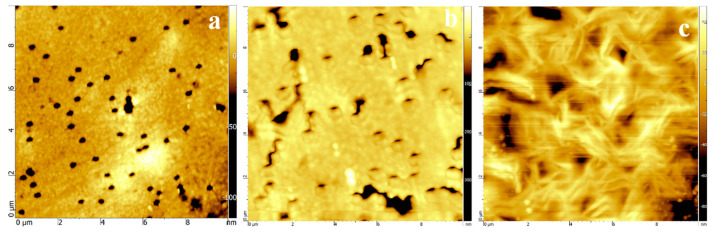
AFM images of original PET TeMs (1 × 10^8^) (**a**), in comparison with SM-grafted PET TeMs at grafting degree of 3.6% (**b**) and 15.1% (**c**) with size of 10 × 10 µm.

**Figure 5:**
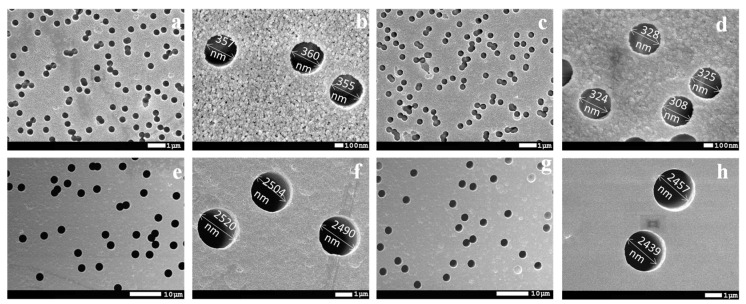
SEM images of original PET TeMs (pore size—350 ± 30 nm, pore density 1 × 10^8^) (**a**,**b**), after modification at optimal conditions (grafting degree—3.6%) (**c**,**d**) original PET TeMs (pore size—2.50 µm, pore density 1 × 10^6^) (**e**,**f**), after modification at optimal conditions (**g**,**h**).

The surface morphology of the membranes before and after modification was studied by AFM and SEM. AFM images are shown in Figure 4, and SEM images are shown in Figure 5. Figure 4 shows that at monomer concentrations of 15%, uncontrolled graft polymerization occurs. Moreover, surface roughness increased from 2.15 nm to 5.14 nm and to 9.57 nm with a grafting degree of 3.6% and 15.1% of SM, respectively.

It is difficult to compare the obtained results on photoinitiated graft polymerization of SM with those available in the literature since there are few data. A similar monomer (lauryl methacrylate (LM)) was used for thermally initiated graft polymerization onto bamboo fiber for use as material for oil spills [31]. Optimal conditions were found to be [AIBN] = 0.04 mol/L, [LM] = 1.0 mol/L, 180 min, and 75 °C. The grafting degree was reached up to 31.28%.

At optimal conditions, PET TeMs with a pore density of 1 × 10^8^, a pore size of 350 nm and PET TeMs with a pore density of 1 × 10^6^, pore sizes of 2.1, 2.5, 2.8 and 3.05 µm were hydrophobized by graft polymerization of SMA at optimal conditions (monomer concentration of 5%, time of UV irradiation is 60 min) and used for oil–water emulsion separation. It should be noted, that for all membranes, CA was almost the same and equal to 109°. From SEM images presented in Figure 5, it is seen that all membranes retain their pore structure; after hydrophobization, only a slight change in surface morphology is observed.

**Figure 6:**
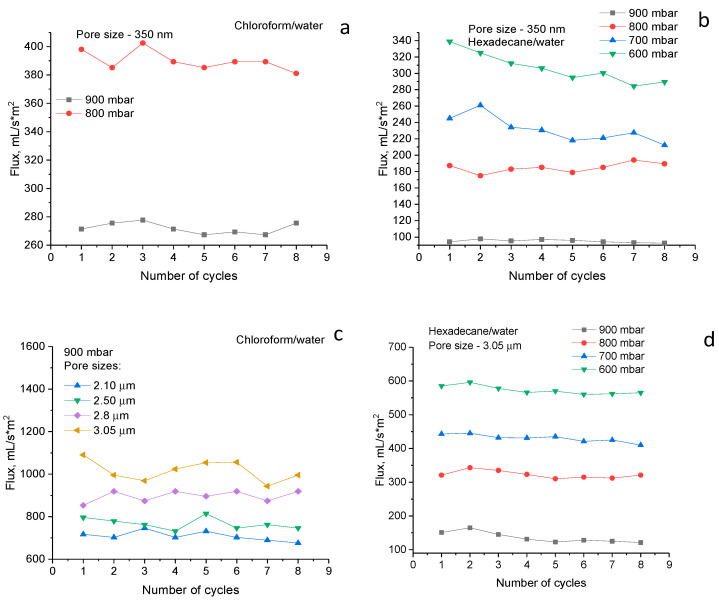
The fluxes of hydrophobic PET TeMs with pore density of 1 × 10^8^ (**a**,**b**) and with pore density of 1 × 10^6^ (**c**,**d**) for chloroform/water (**a**,**c**) and hexadecane/water (**b**,**d**) emulsions during 8 cycles of testing.

The results of measuring the performance of SM grafted PET TeMs with different pore diameters by using chloroform–water and hexadecane–water emulsions are shown in Figure 6. Primarily, membrane testing was carried out at a pressure difference of 900 mbar. If the flux was satisfactory, then reducing the pressure would not make sense. However, when using membranes with a pore density of 1 × 10^8^ pores/cm^2^ and when using a hexadecane–water emulsion, the fluxes were unsatisfactory, and in order to increase fluxes, the effect of pressure on flux was studied. There is a significantly greater flux in all cases for chloroform–water than hexadecane–water emulsion, which can be associated with the higher viscosity of hexadecane–water emulsion in comparison with chloroform–water emulsion. With an increase in the pore diameter, a regular increase in fluxes is observed and this parameter reaches a maximum value of 1100 mL/m^2^∙s with a pore size of 3.05 µm and a pressure of 900 mbar. The degree of purification varies in a narrow range (99.8–97.0%). The stability of the hydrophobic membrane was studied for 8 cycles. Only a slight decrease in fluxes is observed, in general, and the membranes remain stable, the degree of separation does not change. In addition, it should be noted that the original PET TeMs were also tested in emulsion filtration; however, separation efficiency was close to zero. It was observed that, first, chloroform passes through the pores of the membrane, and then the water is filtered out. Thus, the developed method of PET TeMs modification can be used for hydrophobization of membranes with large pores and has successfully been used for oil–water separation.

## 4. Conclusions

In this study, we presented the results of PET TeMs hydrophobization by photo-induced graft polymerization of stearyl methacrylate. The effect of monomer concentration and time of UV irradiation was studied, and optimal conditions leading to the highest water contact angle with minimal change in pore structure were found. PET TeMs with pore diameters ranging from 350 nm (pore density of 1 × 10^8^) to 3.05 µm (pore density of 1 × 10^6^) were tested in oil–water emulsion separation by using hexadecane–water and chloroform–water model systems as emulsions. Membranes have shown stable fluxes and separation degrees during eight filtration cycles for emulsion separation. Membranes with large pore diameters showed a maximum flux value of 1100 mL/m^2^∙s for chloroform–water emulsion at a vacuum pressure of 900 mbar and 573 mL/m^2^∙s for hexadecane–water emulsion at a vacuum pressure of 600 mbar. The increase in fluxes for hydrophobic membranes with a pore size of 3.05 μm and a pore density of 1 × 10^6^ was almost three times greater than for membranes with a pore size of 350 nm and a pore density of 1 × 10^8^.

## Figures and Tables

**Table 1:** Main parameters of PET TeMs before and after grafting with SM.

Time of Irradiation, Min	Monomer Concentration, %	Degree of Grafting, %	Effective Pore Diameter, nm
**0**	-	-	350 ± 30 *
30	5	2.4	326 ± 5
45	5	2.9	322 ± 4
60	5	3.6	319 ± 5
90	5	7.1	283 ± 4
120	5	15.1	226 ± 2
60	1	0	345 ± 5
60	2.5	2.8	321 ± 5
60	10	10.7	216 ± 6
60	15	53.1	-
			
**0**	-	-	3050 ± 30 **
30	5	2.3	3055 ± 25
45	5	2.7	2995 ± 28
60	5	3.4	2984 ± 26
90	5	6.5	2910 ± 25
120	5	14.0	2920 ± 32
60	1	0	3040 ± 28
60	2.5	2.4	2990 ± 25
60	10	10.8	2935 ± 29
60	15	45.2	2746 ± 35
60	5	3.40	2095 ± 25 ***
60	5	3.35	2465 ± 32 ***
60	5	3.51	2785 ± 30 ***

*—pore density of 1 × 10^8^; **—pore density of 1 × 10^6^; ***—initial PET TeMs with pore density of 1 × 10^6^ and pore diameter of 2.10, 2.50, 2.80 µm, respectively.

**Table 2:** Change in the contact angle and surface free energy contributions of membranes as a function of the time of UV irradiation and concentration of monomer (pore diameter of pristine PET TeMs is 3.05 µm, pore density is 1 × 10^6^ pore/cm^2^).

Time, Min	Concentration, %	Degree of Grafting, %	θ, Water	θ, Diiodomethane	γ, mJ/m^2^	γ_p_, mJ/m^2^
0	-	-	80.1	25.7	48.3	2.4
30	5%	2.3	99.6	53.0	32.9	0.3
60	5%	3.4	109.0	71.1	22.4	0.1
90	5%	6.5	99.1	54.8	32.0	0.4
120	5%	14.0	99.4	39.7	39.8	0.1
60	10%	10.8	99.7	61.1	28.6	0.6
60	15%	45.2	94.0	30.3	28.8	1.5

## Data Availability

The data presented in this study are available on request from the corresponding author.
